# First Clinical Evidence About the Use of a New Silver-Coated Titanium Alloy Instrumentation to Counteract Surgical Site Infection at the Spine Level

**DOI:** 10.3390/jfb16010030

**Published:** 2025-01-16

**Authors:** Lucrezia Leggi, Silvia Terzi, Maria Sartori, Francesca Salamanna, Luca Boriani, Emanuela Asunis, Cristiana Griffoni, Gianluca Giavaresi, Alessandro Gasbarrini

**Affiliations:** 1 Spine Surgery, IRCCS—Istituto Ortopedico Rizzoli, Via G.C. Pupilli 1, 40136 Bologna, Italy; lucrezia.leggi@ior.it (L.L.); silvia.terzi@ior.it (S.T.); luca.boriani@ior.it (L.B.); emanuela.asunis@ior.it (E.A.); cristiana.griffoni@ior.it (C.G.); alessandro.gasbarrini@ior.it (A.G.); 2 Surgical Sciences and Technologies, IRCCS—Istituto Ortopedico Rizzoli, Via di Barbiano 1/10, 40136 Bologna, Italy; francesca.salamanna@ior.it (F.S.); gianluca.giavaresi@ior.it (G.G.); 3 Department of Biomedical and Neuromotor Sciences, University of Bologna, Via Massarenti 9, 40128 Bologna, Italy

**Keywords:** surgical site infection, spinal surgery, silver-coated instrumentation

## Abstract

**Background:** Surgical site infections (SSIs) following spinal instrumentation surgery are among the most concerning complications. This study is aimed at assessing the effectiveness of a new treatment approach for SSIs that includes a single-stage approach with the removal of the previous hardware, accurate debridement, and single-stage instrumentation using a silver fixation system (SFS) made of titanium alloy coated with silver (Norm Medical, Ankara, Turkey) by means of a retrospective observational study. **Materials and Methods:** The demographic data, type of surgery, comorbidities, pathogens, and treatment details of consecutive patients with an SSI who received the SFS between 2018 and 2021 were extracted from their medical records and analyzed. The patients treated with the SFS for primary pyogenic infections were excluded. The patients were re-evaluated at multiple endpoints in order to assess the rate of reinfection and the local and general complications. **Results:** Fifty-six patients were treated with the SFS and thirty-four patients met the inclusion criteria. Out of those 34 patients, the rate of infection recurrence or insurgence after the implantation of the SFS was 11.8%, with infection detected in 4 out of 34 cases and mechanical problems detected in 2 of the 34 cases (5.9%). The overall success rate in controlling infection recurrence or emergence was 88.2% (30 out of 34 cases). The overall survival rate of the SFS was 87%, 78%, and 71% at one, two, and three years, respectively. **Conclusions:** The surgical strategy with the SFS demonstrated promising outcomes in preventing infection recurrence or insurgence, with a low incidence of mechanical complications. However, further structured and comprehensive studies are essential for validating these initial findings.

## 1. Introduction

In vertebral surgery, the complexity of the pathologies and the surgical procedures and the involvement of critical anatomical structures further increase the potential array of complications, which range between 7% and 20% according to the literature data [1]. Among the complications, surgical site infections (SSIs) represent a growing concern, ranking as the third most commonly encountered cause [2]. The incidence varies depending on factors such as the patient’s diagnosis, the characteristics, the procedure complexity, the operating surgeon, and several other variables. Furthermore, the use of instrumentation in the treatment of spinal pathologies increases the risk of infection. Currently, there is no shared accepted protocol for managing SSIs involving spinal instrumentations [3,4], which are an essential component of several spinal disease treatments. Additionally, treating SSIs of the spine is particularly challenging owing to the unique characteristics and biomechanics of the spine [5]. The standard approaches often fall short in addressing infections related to instrumentation. The formation of biofilms considerably complicates the treatment options and the likelihood of completely eradicating the infection [6]. For early infections, a “conservative” approach including debridement, antibiotic therapy, and implant retention (DAIR) can be attempted. Moreover, wound management techniques, such as primary wound closure with the use of suction drains, closed suction irrigation systems, and vacuum-assisted wound closure (VAC), are employed [7,8]. However, in chronic infections, implant loosening frequently results in mechanical instability and the presence of biofilm considerably reduces the efficacy of DAIR. In such cases, the only available option is an implant revision, which involves replacing the existing instrumentation with new implants [9].

The presence of biofilms on implants presents significant challenges, leading to extensive research into surface functionalization in an attempt to develop devices capable of resisting microbial colonization [10]. Silver has emerged as a strong candidate for device coatings due to its broad-spectrum antibacterial activity, effective against both Gram-positive and Gram-negative bacteria, including multidrug-resistant strains. Silver possesses bacteriostatic, bactericidal, and antifungal properties derived from several mechanisms of action [11,12,13].

Owing to its unique properties, silver in various forms (ionic, colloidal, combined, or nanoparticle form) has found several applications as a coating for different biomedical devices, such as vascular prostheses and catheters, as well as in wound management [14]. The use of silver, along with other antimicrobial agents, aligns with the concept of creating implants and devices capable of defending themselves against pathogens, thereby reducing the reliance on antibiotics. While the evidence of silver’s antibacterial activity in preclinical models has been established, its application in the orthopedic field remains limited [15,16,17,18] and, to the best of our knowledge, there is no study in the literature specifically focusing on the evaluation of the performance of silver coating in spinal surgery. Considering this, a new silver-coated instrumentation was investigated in a specific cohort of patients affected by SSIs or at high risk of infection, with the aim of assessing the effectiveness and safety of this new approach in preventing/treating SSIs following spinal instrumentation surgery.

## 2. Materials and Methods

The new antibacterial spinal system (Silver Posterior Thoracolumbar Fixation System—SFS) is manufactured by Norm Medical (Ankara, Turkey) and all components are made of titanium alloy (Ti_6_Al_4_V) coated with a micrometric silver coating obtained through a sol–gel deposition technique. This instrumentation was adopted to treat primary spinal infection (spondylodiscitis) or when an SSI was diagnosed or suspected in patients who had previously had instrumentation implanted, in accordance with a panel of clinical, laboratory, and diagnostic criteria (i.e., erythema or swelling of the incision in the case of previous surgery, wound dehiscence and/or purulent drainage from the wound, worsening local pain, fever, an elevated erythrocyte sedimentation rate (ESR) and/or C-reactive protein (CRP), and a positive PET-CT or MRI [19]).

A retrospective observational study was conducted in order to assess the effectiveness of a Silver Fixation System (SFS) in preventing the recurrence or emergence of SSIs. The retrospective study received ethical approval from the Ethical Committee of Area Vasta Emilia Centro (AVEC) under the protocol number 232/2021/Oss/IOR. The inclusion criteria for the study were as follows: age more than 18 years; the presence of a surgical site infection or a late surgical site infection (>30 days) or clinical suspicion of SSI recurrence following a previous spinal surgery; the replacement of the pre-existing instrumentation with the SFS; and a minimum follow-up period of 6 months after the surgery with the SFS. The exclusion criteria were as follows: treatment with the SFS for a primary infection (spondylodiscitis); the occurrence of early surgical site infection (<30 days); and a follow-up inferior to 6 months in the case of treatment with the SFS for surgical site infection.

Study-specific informed consent was not required for this retrospective observational study due to the regulations relevant to health institutions dedicated to scientific research.

All of the spinal procedures were performed at a tertiary reference center for spine surgery between January 2018 and June 2021. The follow-ups were collected up until December 2022. The decision to use the new silver-coated instrumentation was made by the spine surgeon when it was technically feasible and when there were no contraindications, as advised by the infectious disease consultants.

During the surgery, the existing instrumentation was removed and sent to the laboratory for sonication and microbiological tests. Multiple tissue samples (at least five) were taken from the surgical site for microbiological and histological investigations. Subsequently, the SFS was implanted. Before the final suturing, meticulous curettage of the soft tissues was performed to remove the necrotic and/or infected tissue, ensuring access to viable, well-perfused tissues. Copious pulsatile saline washes (5–10 L) were administered, followed by a thorough hemostasis. A subfascial suction drain was placed, and the wound was closed with a monofilament thread.

The postoperative hospital stay involved a two-day observation period in the postoperative intensive care unit. Following this, the patients started a rehabilitation program aimed at restoring the ability to stand and walk independently. From the immediate postoperative period, patients underwent broad-spectrum antibiotic therapy, which was subsequently modified according to their microbiological cultures and antibiogram results.

Upon discharge, the patients received instructions regarding therapy, wound management, rehabilitation, and follow-ups. Wound care was generally performed twice a week, and the suture was removed at 20 days after surgery. Weekly blood tests including a complete blood count with leukocyte formula, inflammation indices, and the assessment of liver and kidney function were performed.

The antibiotic therapy was generally administered for a minimum of 8–10 weeks postoperatively. The follow-up evaluations occurred at six weeks after surgery in a dedicated outpatient clinic, where both an orthopedic specialist and an infectivologist assessed the patient for local and systemic complications. The subsequent evaluations occurred at the completion of the antibiotic therapy and then every six months.

The comorbidity conditions were identified for each patient through the ICD-9-CM diagnosis codes reported in their medical records and evaluated using the Sharma et al. (2021) Swiss weights modification of the Elixhauser comorbidity index (ECI) [20,21], which encompasses 31 individual conditions. The patient’s overall score is calculated by summing up each comorbidity condition, which is assigned a weight based on its association with in-hospital mortality, length of stay, and hospital charges. The Elixhauser weights range from −7 to +17. A score of zero indicates the absence of comorbidities. The higher the score, the greater the likelihood of higher mortality or resource utilization. The ECI scores were further stratified into classes <0, 0, 1–4, and ≥5.

### Statistical Analysis

The statistical analysis was carried out using R software (v.4.3.1) [22], with the use of the “Survival Analysis” (“survival” v. 3.5-7) and the ”Subdistribution Analysis of Competing Risks” (“cmprsk” version 2.2-11) packages [23,24,25]. The demographic and clinical data were reported as the mean and the 95% confidence interval (CI) (continuous variables) or as the frequency (%). The survival analysis was performed using Kaplan–Meier analysis with SFS failure as the endpoint, considered as the need for surgical intervention to remove the instrumentation for the mobilization of or a break in the SFS components or to debride the surgical site for a suspected recurrent infectious event. The SFS survival times of the cases that did not fail were considered at the last observation date (the date of the last follow-up examination or the date of death until December 2022). A Cox regression analysis adjusted for sex and age in classes was performed to identify the variables that might influence SFS survival and to define the hazard ratio of the event (any treatment involving surgery, e.g., DAIR). Finally, the Fine and Gray model was used to investigate if death might represent a competing risk for SFS survivorship by using the cumulative incidence function (CIF) rather than the survival function, which represents the probability of observing a specific type of event before a given time [24,25]. A 5% level of significance was considered.

## 3. Results

### 3.1. Clinical and Demographic Data of Patients Before the SFS Implant

**Figure 1:**
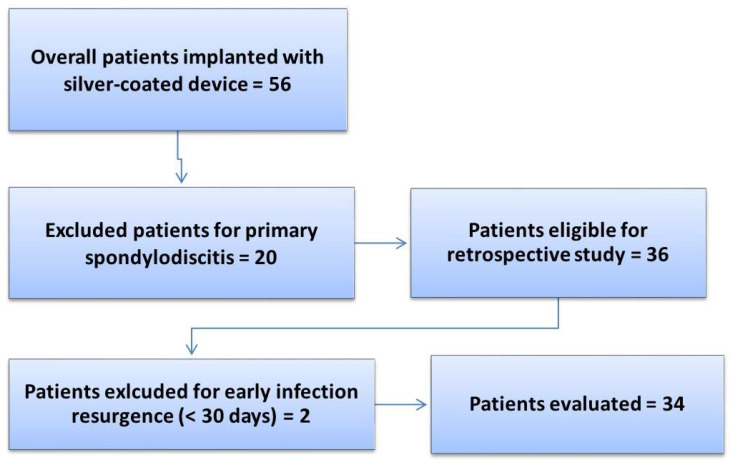
Flowchart of the retrospective study on the SFS.

A total of 56 patients were treated with the silver antibacterial spinal system and monitored during the follow-up period (2018–2022) until SFS failure or until December 2022. After applying the inclusion and exclusion criteria for the study, a total of 34 patients with a mean age of 59 (95% CI [55, 63]) years were included in the final analysis. The exclusion details for the patients are reported in Figure 1, while Table 1 reports the clinical and demographic data of the patients included in the study.

No significant differences were observed among the different age classes, while significant differences were identified in the BMI classes, with the highest percentages recorded in the “healthy weight” and “overweight” classes (38%, *p* < 0.005). Additionally, the number of non-smokers was significantly higher compared to the number of smokers (71%, *p* < 0.05). Nineteen patients (56%) were treated for nononcologic spinal diseases and fifteen for spinal tumors (44%), with no significant difference between the two patient subgroups as far as the gender distribution was concerned. The last surgical treatment before the SFS implantation was reported as follows: 14 patients (41%) had undergone a previous revision surgery, 11 patients (32%) had undergone primary stabilization surgery, and nine patients (26%) were submitted to “en bloc” resection (vertebrectomy) for spinal tumors. Thirteen patients (38%) had received a previous surgical treatment for postoperative infection at an average of 112 (95% CI [70, 154]) days after surgery. Of the 13 patients previously treated for postoperative infection, 10 underwent surgical debridement while the remaining 3 underwent a spinal fixation removal. No significant gender differences were observed for these variables (Table 1).

**Figure 2:**
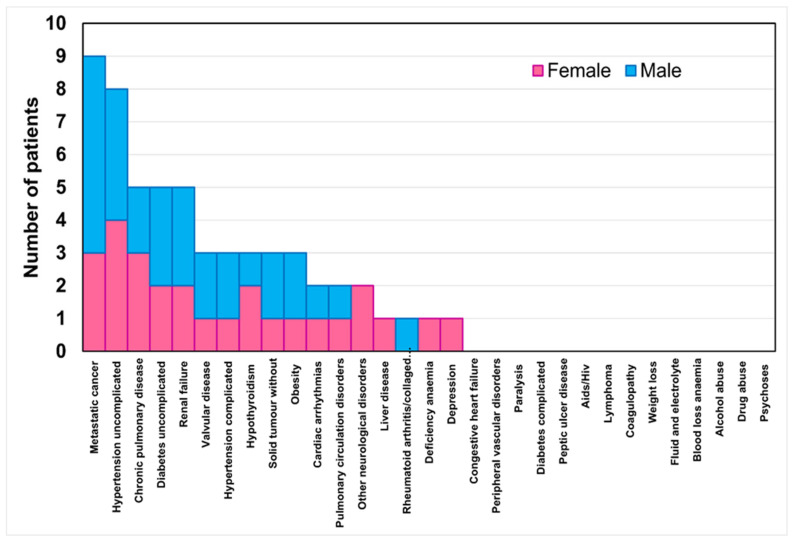
Stacked histogram of the number of patients per sex for the comorbidity classes in the Elixhauser comorbidity index.

Figure 2 reports the stacked histogram of the number of patients presenting comorbidities classified by the Elixhauser comorbidity index. In total, 53% of the women and 41% of the men had at least one comorbidity. This percentage decreased to 35% of the women and men with two comorbidities, then to 29% of the women and men with three comorbidities, and finally to 15% of the women and men with four comorbidities. No significant differences were found between the sexes concerning the number of comorbidities present.

### 3.2. Clinical Records from the SFS Implant

Of the patients having surgery with the SFS, 19 (10 females and 9 males) were receiving antibiotics for previous surgical site infections (SSIs). Among these, the most commonly used combination was Daptomycin in association with Fosfomycin or with Piperalillin + Tazobactam or Rifampin + Levofloxacin.

**Figure A1:**
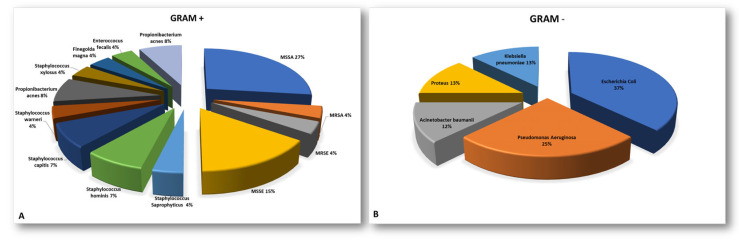
Pie charts representative of the pathogens isolated from instrumentations or tissue biopsies during revision surgery split between Gram-positive (**A**) and Gram-negative (**B**). (MRSA = methicillin-tesistant Staphylococcus aureus; MSSA = methicillin-sensitive Staphylococcus aureus; MRSE = methicillin-resistant Staphylococcus epidermidis; MSSE = methicillin-sensitive Staphylococcus epidermidis).

A microbiological culture isolation from the sonicated instrumentation or tissue samples revealed or confirmed SSIs caused by a single microorganism in 17 of the 34 cases (50%) and SSIs caused by multiple microorganisms in 7 out of 34 cases (21%), and negative culture results were obtained in 10 out of 34 patients (29%), despite a clinical suspicion of infection (*χ*² = 4.64, *p* = 0.098). The Staphylococcaceae family was the most commonly isolated group among the Gram-positive bacteria (79%). Meanwhile, Escherichia Coli (40%) was the most prevalent pathogen among the Gram-negative bacteria. More information can be found in Appendix A in Figure A1 and its caption.

In the postoperative period following the SFS surgery, the patients were given antibiotic treatment as prescribed by the infectious disease specialists and based on the antibiograms obtained from the post-culture isolation, as shown by the data reported in Table A1. The average duration of treatment after surgery was 2.8 95% (95% CI [2.4, 3.2]) months.

According to the surgical site where the SFS was implanted, the surgeries were classified as thoracic (20.7%), lumbar (8.8%), thoracolumbar (23.5%), lumbosacral (32.3%), or thoraco-lumbosacral (14.7%), as detailed in Table 2. In the early postoperative period, three general clinical complications related to hospitalization occurred: one case of urinary tract infection, one case of deep vein thrombosis, and one case of diverticulitis. Unfortunately, a 66-year-old woman died 10 days after undergoing lumbosacral SFS surgery as a result of the deterioration of her clinical condition, which was severely compromised by pre-operative sepsis.

The length of stay was 15 days (95% CI [11, 19]) and the healing time after the SFS surgery was 4.2 months (95% CI [3.1, 5.3]), without any differences between sexes.

Complications occurred in 8 cases out of 34 patients treated with the SFS (23.5%). In total, 2 out of 34 cases (5.9%) were treated with a DAIR approach due to a suspected infection recurrence after the SFS implantation, as described in Appendix A (Complications Description paragraph).

In total, 4 cases out of 34 (11.7%) necessitated the removal of the SFS for infection recurrence (in Appendix A, a detailed description of each case is reported).

The revision of the SFS instrumentation was required in another 2 of the 34 cases (5.9%) due to mechanical problems, such as screws loosening or rod breakage. These issues arose 18 months and 27 months after the initial SFS surgery. Finally, no clinical signs of argyria were reported during the follow-up period.

At follow up visits, the quality of life of patients was evaluated together with clinical assessment and resulted to be improved in relation to the favorable clinical outcome of the surgery and the healing of the infection.

**Figure 3:**
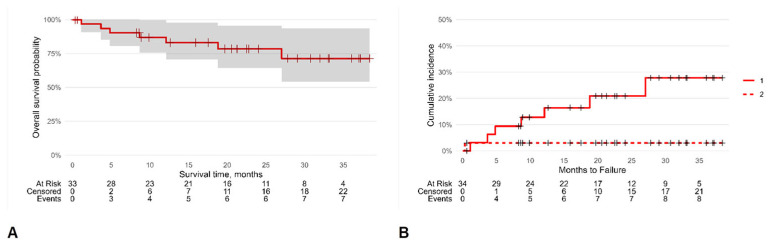
Kaplan–Meier survival analysis: overall survival probability (**A**) and cumulative hazard incidence (**B**). The symbol + represents censored patients; continuous line (1) is the cumulative hazard incidence of censored patients while dashed line (2) is that for dead patients.

A Kaplan–Meier analysis was carried out to ascertain the chances of SFS survival until failure (Figure 3). The survival rate overall was 87% (95% CI [76, 100]), 78% (95% CI [64, 96]), and 71% (95% CI [54, 94]) at one, two, and three years, respectively, with an incidence density of 0.14 SFS failures/person-year.

The selected multivariate Cox regression model (the lowest AIC value) was adjusted for sex and age, and the competing risk of patient mortality on SFS survival was considered according to Fine and Gray’s model. This analysis demonstrated that the HR of SFS failure was 8.26 (95% CI [1.05, 65.0], *p* = 0.045) for the patients with a recurrent infection that was surgically treated before the SFS implantation and 6.97 (95% CI [1.99, 24.4], *p* = 0.002) for the patients undergoing vertebrectomy rather than arthrodesis or revision surgery (Table 3).

## 4. Discussion

In our ongoing study, we assessed the efficacy of a novel SFS in association with antibiotic therapy for the treatment and prevention of infection recurrence or development in cases where implant retention was not feasible and re-instrumentation was necessary. This study presents a strong element of novelty, as all of the included patients underwent re-instrumentation with the SFS due to an active infection or based on diagnostic criteria suggesting the probable presence of infection. Among the 34 patients instrumented with the SFS, 4 cases out of 34 (11.7%) required further SFS revision because of the resurgence or suspected relapse of infection. In another 2 cases out of 34 (5.9%), the DAIR approach proved sufficient to manage the complication, while an additional 2 patients required revisions due to mechanical issues (screw mobilization and rod breakage) (5.9%). The overall success rate in controlling infection relapse or emergence was 88.2% (30 out of 34 cases), while the rate of infection recurrence or insurgence was 11.7%, with infection detected in 4 out of 34 cases.

Owing to the unique nature of our intervention, comparisons with similar literature data are challenging. In addition, despite the extensive preclinical data on antibacterial strategies for orthopedic devices [26,27], very few approaches or innovations effectively translate into clinical applications, and even fewer are available in the field of spinal surgery. To the best of the authors’ knowledge, the data presented in this paper represent one of the few clinical pieces of evidence regarding the use of silver-coated instrumentation in spinal procedures. Only one other clinical study has been published, where the authors examined the impact of silver-coated transpedicular stabilization devices on renal and/or hepatic function and on serum silver concentration in 50 patients. The authors reported no complications or infection insurgence or recurrence after a one-year follow-up. No changes were detected in the renal and hepatic values. Moreover, the levels of silver in the urine and serum were undetectable at the point of each sampling time (10th day and 1st, 3rd, 6th, and 12th month). Furthermore, the authors reported no complications or implant infections associated with the instrumentation [28]. An additional clinical study has been performed regarding the spine, which focused on the examination of a silver-containing hydroxyapatite-coated lumbar interbody cage. In this study, the aim was to explore the potential occurrence of adverse events associated with silver and to assess the level of bone fusion in the patients undergoing posterior lumbar interbody fusion surgery. However, this study involved a cohort of patients diagnosed with degenerative spinal conditions, with no reference to infection or the risk of infection development among the inclusion criteria [29]. Several studies have investigated iodine-coated titanium devices as an alternative antimicrobial strategy for treating or preventing infections in compromised patients. One study involving 222 patients treated with iodine-coated devices—including spinal instrumentation but also osteosynthesis plates, prostheses, and nails—reported an infection rate of 1.9% (3 out of 158 patients) among those treated prophylactically, and no infection reactivation was observed in the group treated for active infections [30]. Another retrospective study, which included different type of coated device, analyzed 72 patients and reported a reinfection rate of 4.2%, with no reinfections associated with the vertebral instrumentation [31]. A third study specifically evaluated iodine-coated spinal implants in 14 patients with pyogenic vertebral osteomyelitis, showing complete infection regression and no adverse effects [32]. Despite these promising results, the aggregated presentation of much of the data—without clear clinical or demographic details or distinctions regarding the anatomical sites—limits the broader interpretation and applicability of these findings.

Therefore, the closest comparable data in the literature come from clinical evidence involving silver-coated megaprostheses [26]. Referring to these data and comparing our results with the performance of the megaprostheses used for revisions in cases of septic complications, the reported performance of PorAg^®^ in preventing infection recurrence was 2 cases out of 21 patients (9.5%) [33]. In the case series analyzed by Wafa et al. using Agluna^®^, in the group treated with silver, infection recurred in 3 out of 20 patients (15%), in comparison to the control group, in which 9 out of 21 patients (42.9%) experienced infection recurrence [34]. Our results showed that among the 34 patients who underwent re-instrumentation with silver, 88.2% (30/34) remained infection free at the time of the last follow-up while 11.7% (4 cases out of 34) experienced infection recurrence, demonstrating that our data align with the performance of devices with similar concepts and application that are already used in clinical practice.

Despite these encouraging results, this study is affected by several limitations. Foremost among them is the absence of a control group consisting of patients who underwent re-instrumentation with standard devices, which represents a significant limitation. The sample size of the included patients is limited, and a prospective randomized clinical study with a homogeneous patient cohort is necessary to draw more robust conclusions. Indeed, our retrospective study encompassed a heterogeneous group of patients in terms of age, comorbidities, associated pathologies, and surgical stabilization levels, leading to increased inter-patient variability. However, it is worth noting that this diverse patient group is representative of the cases typically referred to tertiary centers for the treatment of spinal pathologies and reflects the high-risk population commonly encountered in clinical practice. Another limitation of our study is the absence of an assessment of the local or serum levels of silver released by the instrumentation’s components. We are aware that the use of silver raises concerns regarding potential adverse effects stemming from the possible accumulation of silver in various tissues and organs. Apart from argyria, which has a reported incidence ranging widely from 0% to 23%, the data from the assessments conducted on megaprostheses have, until now, not shown any local or general side effects in humans [35]. This is despite the testing of various silver coatings, even with a declared silver content higher than that employed in our spinal instrumentations, as seen with Agluna^®^ (stated as 6 mg to coat Agluna^®^ compared to the 2–5 mg declared for Normed Silver). A recent systematic review and meta-analysis performed on the main system available on the market indicated an overall infection rate of 9.2% for silver-coated megaprostheses compared to 11.2% for uncoated devices, with a lower reinfection rate observed for silver-coated implants used in revision surgery (13.7%) in comparison to uncoated prostheses (29.2%) [11,35].

Despite the limitations, this study represents the first clinical report on the use of an SFS in the spinal district, where managing infections proves to be even more complex compared to other anatomical sites. Out of 34 patients, the infectious event was successfully treated or prevented in 30 patients at the last available follow-up, yielding a success rate of approximately 88%. This outcome was achieved through the combination of the SFS instrumentation with an appropriate antibiotic therapy tailored based on the identified pathogen or the patient’s clinical history. Particularly noteworthy is the survival of the instrumentation itself, with an overall survival rate of 87%, 78% and 71% at one, two, and three years, respectively, as well as a low percentage of mechanical complications.

Thoroughly investigating the long-term safety profile of silver instrumentation, its functional outcome, and its comparative effectiveness by constructing more robust clinical studies, as well as foreseeing specific investigations, is certainly necessary and warranted to solidify the role of the SFS in clinical practice. Nevertheless, these preliminary results are very encouraging, suggesting the potential use of an additional tool in the fight against infections.

## 5. Conclusions

The results of the present study suggest that the SFS may offer significant utility in managing high-risk or infected patients by reducing the incidence or recurrence of infections. In our study, we were able to prevent infection onset or recurrence in 30 out of 34 patients (88.2%), with only 4 cases of infection recurrence (11.7%). Nevertheless, it is imperative to establish a comprehensive long-term monitoring system for silver serum levels to ensure safety and optimize effectiveness. Despite the limitations, our study represents a pivotal starting point with substantial clinical relevance in the literature. It paves the way for an innovative approach to late post-surgical infections in spinal surgery, as it stands as the inaugural study of its kind.

## Figures and Tables

**Table 1:** Demographic and clinical data of the patients included in the study before the treatment with the Silver Fixation System.

Variables		Total	*p*-Value	Female	Male	*p*-Value
Patients (n)		34		15	19	0.398
Age (yrs)		59 [55, 63]		58 [53, 63]	59 [55, 63]	0.608
Age class, n (%)	≤40	4 (11.8)	0.159	2 (5.9)	2 (5.9)	0.923
	41–50	4 (11.8)		2 (5.9)	2 (5.9)	
	51–60	8 (23.4)		4 (11.7)	4 (11.7)	
	61–70	12 (35.2)		4 (11.7)	8 (23.5)	
	>70	6 (17.8)		3 (8.9)	3 (8.9)	
BMI class, n (%)	Underweight <18.5	2 (5.8)	<0.005	1 (2.9)	1 (2.9)	0.943
	Healthy weight 18.5–24.9	13 (38.3)		6 (17.7)	7 (20.6)	
	Overweight 25.0–29.9	13 (38.3)		6 (17.7)	7 (20.6)	
	Class 1 obesity 30.0–34.9	4 (11.8)		1 (2.9)	3 (8.9)	
	Class 2 obesity 35.0–39.9	2 (5.8)		1 (2.9)	1 (2.9)	
Smoking, n (%)	N	24 (70.6)	0.016	11 (32.4)	13 (38.2)	1.000
	Y	10 (29.4)		4 (11.8)	6 (17.6)	
Oncological diseases, n (%)	N	19 (55.9)	0.493	10 (29.4)	9 (26.5)	0.314
	Y	15 (44.1)		5 (14.7)	10 (29.4)	
Type of surgery before SFS, n (%)	Arthrodesis	11 (32.4)	0.572	4 (11.8)	7 (20.6)	0.657
	Revision surgery	14 (41.2)		9 (26.4)	5 (14.7)	
	Vertebrectomy	9 (26.4)		2 (5.9)	7 (20.6)	
Treatment for infection before SFS, n (%)	Spinal fixation removal	3 (23.0)	< 0.005	1 (7.7)	2 (15.4)	0.842
	Surgical debridement	10 (77.0)		4 (30.7)	6 (46.2)	

**Table 2:** Clinical data related to the treatment with the Silver Fixation System.

		Total	*p*-Value	Female	Male	*p*-Value
Patients (n)		34		15	19	
Site, n (%)	Thoracic	7 (20.7)	0.248	3 (8.8)	4 (11.9)	0.739
	Lumbar	3 (8.8)		2 (5.9)	1 (2.9)	
	Thoracolumbar	8 (23.5)		2 (5.9)	6 (17.6)	
	Lumbosacral	11 (32.3)		5 (14.7)	6 (17.6)	
	Thoraco-lumbosacral	5 (14.7)		3 (8.8)	2 (5.9)	
Complications, n (%)	No	26 (76.6)	<0.0005	11 (32.4)	15 (44.2)	0.929
	Mobilization/break of SFS components	2 (5.9)		1 (2.9)	1 (2.9)	
	Suspected or relapsing infective event	6 (17.6)		3 (8.8)	3 (8.8	
LOS (days)		15 [11, 19]	-	14 [10, 18]	17 [10, 23]	0.559
Healing time after SFS (months)		4.2 [3.1, 5.3]	-	4.0 [2.3, 5.7]	4.4 [2.8, 6.0]	0.715
Life SFS (months)		19.1 [15.8, 22.4]	-	15.8 [10.8, 20.8]	21.6 [17.4, 25.9]	0.067

**Table 3:** Comorbidities of patients treated with the Silver Fixation System expressed as the Elixhauser comorbidity index (ECI).

	Score	*p*-Value	<0	0	1 to 4	≥5	*p*-Value
N	7 [4, 10]	-	8	5	2	19	-
Sex (n)							
Female	7 [2, 12]	0.954	3	2	2	8	0.536
Male	7 [3, 10]		5	3	-	11	
Age (n)							
≤40	5 [−2, 12]	0.592	0	2	1	1	0.167
41–50	1 [−3, 4]		2	1	0	1	
51–60	9 [1, 16]		2	0	0	6	
61–70	10 [4, 15]		2	2	0	8	
>70	5 [1, 10]		2	0	1	3	
BMI (n)							
Underweight <18.5	2 [−23, 26]	0.795	1	0	0	1	0.502
Healthy weight 18.5–24.9	7 [4, 11]		2	3	0	8	
Overweight 25.0–29.9	8 [3, 14]		3	1	1	8	
Class 1 obesity 30.0–34.9	2 [−7, 10]		2	1	0	1	
Class 2 obesity 35.0–39.9	9 [−3, 21]		0	0	1	1	
LOS (days)							
-			19 [6, 32]	13 [9, 17]	11 [5, 16]	15 [10, 20]	0.818
Healing time after SFS (months)							
-			5 [3, 7]	4 [1, 9]	8 [1, 17]	2 [2, 5]	0.509
SFS failure							
Y	7 [3, 10]	0.996	6	5	1	14	0.700
N	7 [0, 13]		2	0	1	4	
SFS life (months)							
-			23.3 [15.2, 3.3]	16.0 [6.7, 25.3]	12.3 [6.3, 8.2]	18.8 [14.6,3.0]	0.582

## Data Availability

The data presented in this study are available on request from the corresponding author.

## Appendix A

**Complications Description**:

In total, 2 out of 34 cases (5.9%) were treated with the DAIR approach due to suspected infection recurrence after the SFS implant. Both procedures were performed at about one-month post-surgery; one case was found positive for Staphylocccus aureus and was treated with Daptomycin in combination with Rifampicin. The second patient, who had a complicated medical history (oncologic patient with several events of infection), underwent surgical debridement of the skin and soft tissues 42 days after the SFS implantation, followed by antibiotic therapy with Daptomycin/Tazocin until the results of the intraoperative microbiological cultures were available, which returned negative for microorganism isolation.

In total, 4 cases out of 34 (11.7%) necessitated the removal of the SFS for infection recurrence. One case occurred five months after the surgery. The removal was prompted by a Corynebacterium spp. infection, which was treated with Teicoplanin and Levofloxacin, with the subsequent addition of Rifampicin. In a second case, wound dehiscence occurred one month after the revision surgery with the SFS, which was treated with the DAIR approach. Minocycline administration was performed to address the positive findings for Candida albicans. However, seven months later, the patient returned for the complete removal of the instrumentation due to spondylodiscitis caused by Candida parapsilosis, Stafilococcus scheleiferi, and Coagulase-negative staphylococci (CoNS). In this case, new instrumentation was not placed due to the significant contamination of the surgical field. In the third case, four months after SFS placement, the patient underwent surgical debridement, instrumentation revision, and a flap procedure due to significant wound dehiscence, instrumentation exposure, and clinical suspicion of infection recurrence. This decision considered the patient’s medical history, which was marked by infective events related to methicillin-sensitive Staphylococcus. Although intraoperative cultures yielded negative results, Teicoplanin therapy was initiated based on the previous instances of infection. The last case required the revision of the SFS eight months after implantation, as wound dehiscence and the exposure of the instrumentation occurred due to a methicillin-resistant Staphylococcus epidermidis infection. The patient was treated with Linezolid.

**Table A1:** List of antibiotics administered before PRE and POST SFS implant.

Antibiotic Theraphy			
Pre-SFS Implant	No. of Patients (%)	Post-SFS Implant	%
Amoxicillin + Clavulanic Acid	1 (3%)	Amoxicillin + Clavulanic Acid	1 (3%)
Amoxicillin + Cotrimoxazole	1 (3%)	Amphotericin B	1 (3%)
Ciprofloxacin	1 (3%)	Dalbavancin + Voriconazole	1 (3%)
Daptomycin + Fosfomycin	2 (6%)	Daptomycin + Piperalillin + Tazobactam	3 (9%)
Daptomycin + Oxacillin	1 (3%)	Daptomycin + Rifampicin + Fosfomycin	1 (3%)
Daptomycin + Piperalillin + Tazobactam	3 (9%)	Levofloxacin + Cefiderocol	1 (3%)
Daptomycin + Rifampin + Levofluoxacin	1 (3%)	Levofluoxacin + Rifampicin	4 (12%)
Ertapenem + Fosfomycin	1 (3%)	Linezolid + Ertapenem + Fosphomycin	1 (3%)
Levofluoxacin + Rifampicin	2 (6%)	Minocycline + Rifampicin	6 (19%)
Macladin	1 (3%)	NA	5 (15%)
Piperalillin + Tazobactam	1 (3%)	Piperalillin + Tazobactam	1 (3%)
Piperalillin + Tazobactam + Linezolid + Ciprofloxacin	1 (3%)	Piperalillin + Tazobactam + Ciprofloxacin	1 (3%)
Teicoplanin	1 (3%)	Piperalillin + Tazobactam + Linezolid + Ciprofloxacin	1 (3%)
Teicoplanin + Ciprofloxacin	1 (3%)	Piperalillin + Tazobactam + Minocycline	1 (3%)
Teicoplanin + Piperalillin + Tazobactam	1 (3%)	Rifampicin	1 (3%)
No treatment	15 (44%)	Teicoplanin	3 (95)
		Trimetoprim + Sulfametoxazolo + Ciprofluoxacin	1 (3%)
		Trimetoprim + Sulfametoxazolo + Rifampicin	1 (3%)
